# P-1455. Socioeconomic and Regional Impact on Influenza Vaccination in Young Children During the COVID-19 Pandemic

**DOI:** 10.1093/ofid/ofae631.1627

**Published:** 2025-01-29

**Authors:** Tawny Saleh, Mary C Cambou, Karin Nielsen-Saines

**Affiliations:** Division of Preventive Medicine, Department of Medicine, David Geffen School of Medicine, University of California, Los Angeles, California, USA, Los Angeles, California; David Geffen School of Medicine University of California, Los Angeles, Los Angeles, California; David Geffen UCLA School of Medicine, Los Angeles, CA

## Abstract

**Background:**

Influenza (flu) vaccination reduces hospitalizations and severe illness, especially in young children particularly vulnerable to complications

**Table 1:**
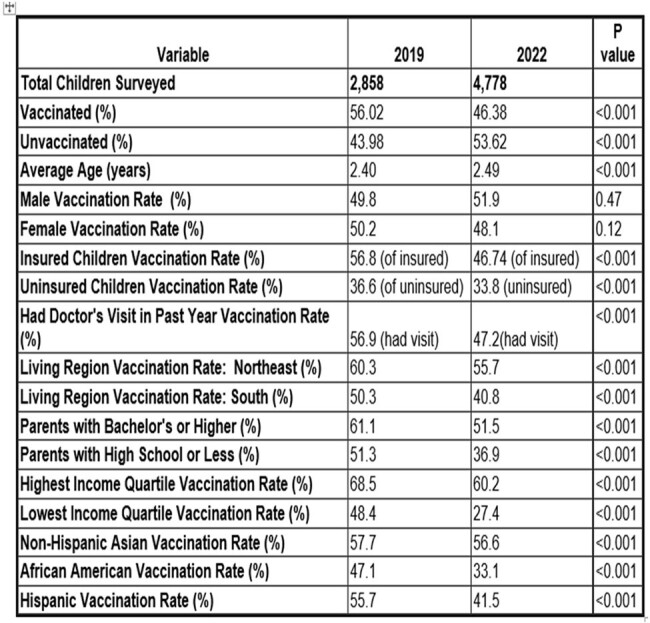
Comparison of Influenza Vaccination Rates and Associated Predictors Among American Children Aged 0-5 Years in 2019 and 2022

This table provides an overview of key variables influencing flu vaccination rates among young children, showing changes between 2019 and 2022. It includes data on the total number of children surveyed, vaccination status, average age, gender distribution, educational levels of parents, family income quartiles, ethnic groups with the highest and lowest vaccination rates, insurance coverage, and whether the child had a doctor's visit in the past year.

**Methods:**

Data from the National Health Interview Survey for 2019 and 2022 were analyzed to assess the impact of parental education, household income, health insurance, and race/ethnicity on flu vaccination rates among U.S. children ≤5 years. Employing logistic regression, Chi-square tests, and Mann-Whitney U tests in STATA, the potential influence of socioeconomic factors on influenza vaccination during the COVID-19 pandemic was evaluated.

**Table 2:**
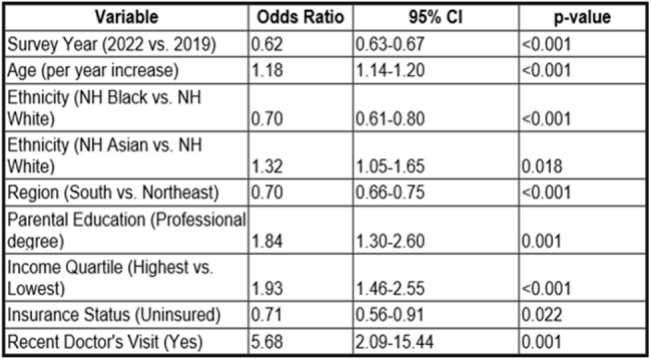
Factors Influencing Influenza Vaccination Rates Among Young Children in the United States, 2019-2022: Results from Logistic Regression Analysis

This table summarizes the logistic regression analysis results, presenting odds ratios, 95% confidence intervals, and p-values for predictors influencing influenza vaccination rates among young children aged 0-5 years. The analysis underscores the significant impact of the survey year, child's age, ethnicity, region, parental education, family income, insurance status, and recent healthcare interactions on vaccination likelihood.

**Results:**

Records for 7636 children in 50 states were reviewed (2858 in 2019 and 4778 in 2022). Flu vaccination rates decreased from 56.02% in 2019 to 46.38% in 2022 (p < 0.001). The decline in flu vaccination rates was most pronounced among younger children, with rates for 2-year-olds reducing from 66.60% to 53.38%, 1-year-olds from 62.95% to 49.03%. and infants from 30.87% to 24.22% (p < 0.001). Insured children were more likely to be vaccinated than uninsured (OR=1.64, p< 0.001). A recent doctor's visit increased the likelihood of receiving a flu vaccine (OR=5.68, p< 0.001). Children from households earning ≥400% of the federal poverty level and those with college or above-educated parents had higher odds of vaccination (OR=1.93, p< 0.001 and OR=1.84, p=0.001, respectively). Significant regional variations in flu vaccination were noted, especially in the Southern U.S. (OR=0.70, p< 0.001). Racial differences were significant, with lower flu vaccination rates among non-Hispanic Black children compared to non-Hispanic Whites (OR=0.70, p< 0.001), while non-Hispanic Asian children exhibited higher flu vaccination rates (OR=1.32, p=0.018).

**Conclusion:**

The decline in flu vaccination rates among young children during the COVID-19 pandemic highlights the influence of socioeconomic factors, healthcare access, and racial disparities on public health outcomes. Addressing these issues is crucial for improving vaccination rates and reducing influenza's impact on young children.

**Disclosures:**

**All Authors**: No reported disclosures

